# Locating Meaning: Health Professionals’ Views on the Psychological and Clinical Significance of Self-Injury Sites

**DOI:** 10.3390/ijerph22070979

**Published:** 2025-06-21

**Authors:** Kathryn Jane Gardner, Rachel Smith, Gillian Rayner, Gary Lamph, Lucie Moores, Robyn Crossan, Laura Bisland, Nicky Danino, Peter Taylor

**Affiliations:** 1 School of Psychology and Humanities, University of Central Lancashire, Preston PR1 2HE, UKlucie.moores3@nhs.net (L.M.); racrossan1@uclan.ac.uk (R.C.);; 2 School of Community Health and Midwifery, University of Central Lancashire, Preston PR1 2HE, UK; 3 School of Nursing and Midwifery, Keele University, Keele ST5 5BG, UK; g.lamph@keele.ac.uk; 4 School of Computer Science, Leeds Trinity University, Leeds LS18 5HD, UK; n.danino@leedstrinity.ac.uk; 5 Division of Psychology & Mental Health, School of Health Sciences, The University of Manchester, Manchester M13 9PL, UK; peter.taylor-2@manchester.ac.uk

**Keywords:** self-injury, self-harm, suicide, risk, body, location, functions, distress

## Abstract

Background: This study explored how health professionals construct clinical and psychological meaning based on the location of self-injury on the body, particularly in relation to concealed or visible injuries and how they might inform attributions about risk, self-injury functions, and distress. Methods: This study used qualitative thematic analysis of semi-structured interviews with 19 health professionals with experience working with self-injury, exploring perceptions and attributions about self-injury in different body locations. Results: Seven themes emerged. In some cases, staff’s attributions aligned with the findings from studies of those who self-injure, such as injuries to areas such as the neck are higher risk. Location was one factor among others, such as injury severity, that staff considered when assessing the risk of infection or suicide. Staff often viewed visible injuries as less risky and attributed them to interpersonal communicative functions, and concealed injuries to intrapersonal factors, though not all staff shared these perspectives. Some staff considered other potential drivers of injury location, including past experiences such as trauma, demographic factors, mental health diagnoses, and exposure to social influences. Some staff described the practical determinants of injury location, such as ease of access, and considered the impact of self-injury location on themselves and their colleagues. Conclusions: Injury location can influence staff perceptions of risk, self-injury functions and distress, underscoring the need for individualized assessment and formulation of each self-injury episode to ensure appropriate risk management. Staff training should be adapted to address injury location to improve understanding, raise awareness of related attributions, and enhance the development of clinical skills. Organizations should support staff in their role due to the potential emotional impact of working with individuals who self-injure and are at risk of suicide. Future research should investigate whether location-based attributions are associated with unintended clinical consequences, such as inaccuracies in risk assessment and formulation.

## 1. Introduction

The reduction of self-harm is integral to successful suicide prevention and features in the National Suicide Prevention Strategy for England [1] and internationally, such as in the US [2]. Self-harm is a well-evidenced risk factor for future suicide [3,4,5], highlighting the importance of staff having a good understanding of the behaviour to facilitate positive interactions with service users and effective clinical management. If self-harm is effectively managed, we can reduce the risk of suicide.

In the UK, NICE (The National Institute for Health and Clinical Excellence) [6] define self-harm as self-poisoning or injury to the body irrespective of intention This definition encompasses methods that directly damage the skin, such as cutting (the most common method of self-harm in UK community settings, 89%) and methods that cause internal damage, such as self-poisoning (the most common method in UK hospital settings, 71%) [7]. The definition also encapsulates self-harm with or without suicidal intent, unlike ‘Non-Suicidal Self Injury’ (NSSI) which excludes self-harm with suicidal intent and excludes self-poisoning as a method, focusing instead on methods of self-injury that cause tissue damage [8]. While the method of self-harm is a clinically significant factor in understanding the differential risk associated with this behaviour [9,10,11,12], less is known about the salience of injury location on the body. In this study, we focused on understanding injury location for direct methods that cause external tissue damage, such as cutting, with or without suicidal intent. Hereafter, we refer to this as ‘self-injury’.

### 1.1. The Psychological and Clinical Significance of Self-Injury Location

The location of self-injury on the body could be clinically or psychologically meaningful to those who self-injure and those providing care. Most empirical studies reporting self-injury locations have adopted a descriptive approach, presenting either range [13] or frequency of specific body locations [14]. Gardner et al. [15] reviewed the literature and concluded that in largely Caucasian Westernized samples, the most common areas are the arms and legs, followed by the torso. They also concluded that these locations are relatively consistent across cultural and ethnic groups, but there are fewer studies in non-Western cultures and non-white ethnicities.

Gardner et al. [15] discussed potential psychological explanations for self-injury location choice, including: (1) functional perspectives, where visible injuries can help meet interpersonal communication needs and concealed injuries manage perceived shame or stigma; (2) psychoanalytic perspectives, which propose that injuries made to specific locations reflect past experiences, help manage self-identity, or serve as symbolic messages to others; and (3) cognitive perspectives, where people may hold location-specific expectancies (i.e., “If I cut my legs, I will feel better and no-one will see”). Empirical studies exploring the link between location and psychological difficulties are scarce. However, one study [16] found that individuals with complex psychological difficulties, such as borderline personality disorder, were more likely to injure multiple sites.

Regarding risk, a handful of empirical studies have explored the link with self-injury location. Individuals presenting to Emergency Departments in Japan with deep cuts to the wrist and forearm (i.e., higher risk) were more likely to injure multiple locations [17], suggesting that location may be part of clinically complex presentation that increases acquired capability for suicide through tolerance to pain at multiple sites, consistent with Joiner’s interpersonal theory of suicide [18]. Similarly, injuries to high-risk locations such as the neck could reflect Joiner’s concept of habituation to fear of death. Consistent with this, several studies have linked self-injury location to suicide risk. Two studies [10,19] of UK hospital presentations of self-harm found that compared to self-poisoning, suicide risk was higher when cutting areas other than the wrist/arm, particularly the neck. Similarly, Finnish adolescents who cut areas other than the upper arm were more likely to make suicide attempts and plan the cutting, compared to adolescents who cut their upper arm [20]. Gardner et al.’s UK study [21] combined the neck with head, forearm, and wrist into a single conceptual ‘visible self-injury’ category, however, finding associations with a reduced likelihood of previous self-injury, current psychiatric treatment, premeditation, and repeat self-injury. Thus, visible self-injury was less complex and clinically risky.

Collectively, studies from different countries suggest that clinical risk may increase for concealed locations, reflecting a more complex clinical and psychological presentation. When considering specific locations, injuries to the neck may increase suicide risk, perhaps reflecting both expected fatal consequences and an acquired capability for suicide through habituation to risk [18]. However, researchers have not yet examined this issue, nor how staff understand, make attributions about, and respond to injuries in specific locations. It is well documented that staff often make assumptions and attributions about self-injury that can influence care [22,23]. Therefore, research exploring whether staff make attributions based on the location of self-injury on the body could improve understanding and inform assessment, formulation, clinical decision-making, and the management of self-injury.

### 1.2. Health Professionals’ Views About Self-Injury Location

The study by Gardner et al. [21] is, to the best of our knowledge, the first to examine whether injury location influences how clinicians manage self-injury. Although concealed cutting was associated with factors suggesting increased clinical risk, Gardner et al. also found that clinicians were less likely to refer episodes of concealed cutting for a psychosocial assessment or psychiatric treatment, as recommended by NICE guidelines [6], though the effect did not persist when adjusting for covariates. Moreover, clinicians may have made more referrals for the visible injury category because it included the neck area, which they may have perceived as higher risk. While studies have yet to explore this potential link, clinically speaking, suicide risk varies by location [19] and areas close to major blood vessels are anatomically risky due to the potential for rapid and fatal blood loss; hence, it stands to reason that staff who understand this might believe such injuries are higher risk, and perhaps attribute them to suicidal rather than non-suicidal intentions. Ample social psychological evidence shows that individuals make causal attributions about self-injury that may affect the staff response. For example, a study [24] that applied Corrigan et al.’s [22] causal attribution model found that people who perceived self-injury as highly risky were more likely to support coercive and segregatory management strategies.

Research has also examined how professionals working with individuals who self-injure attribute the behaviour to different functions, broadly categorized as interpersonal (e.g., eliciting support) or intrapersonal functions e.g., [25,26,27]. Youth justice staff view self-injury as less serious and less related to emotional distress or suicide risk when attributed to interpersonal than intrapersonal functions [28]. Health professionals have also been found to minimize the seriousness of self-injury, often viewing it as “attention seeking” [29,30,31]. Young people similarly use this term to describe injuries in visible locations [32,33]. However, it is a pejorative label that stigmatizes visible self-injury, rather than recognizing it as a coping strategy, an expression of intense emotions, and a behavioral form of communication, as suggested by a lived experience study linking arm cutting (a more visible area) to the interpersonal function of “a cry for help” [20]. In contrast, Gardner et al. [15] argue that individuals may choose concealed areas (e.g., the upper arm, thighs, or abdomen) to avoid public stigma and the self-injury cycle of shame [34], while injuries to areas such as the genitals may, for some individuals, relate to experiences of abuse. It is important, therefore, that staff recognize that injury location may reflect complex psychological functions. Yet, empirical studies exploring staff understanding of and reactions to self-injury location, and whether they make location-based attributions are lacking.

To summarize, injury location might influence staff reactions and prompt staff to make attributions about self-injury, concerning factors such as risk, function, and distress. Attributions may be inaccurate and misalign with the lived experience of those who self-injure. When people make attributions such as “visible injuries are attention seeking”, they may also experience emotions such as feelings of anger or anxiety, leading to negative interactions with service users during what Rayner and colleagues [35] describe as an interpersonal cycle of self-injury. Distress, as well as function, can vary across episodes of self-injury, hence, NICE [6] recommends that clinicians view each episode as unique and open to interpretation. NICE also advise against global risk stratification into low, moderate, or high risk, since this type of assessment cannot accurately predict the risk of self-injury or suicide [6]. Ultimately, overgeneralized clinical judgements based on presenting features such as injury location are broad, sweeping, unreliable statements that can contribute to negative interactions with clients and mismanagement of self-injury, rather than appropriate care [6,35].

Staff can, however, incorporate injury location into an individualized, dynamic, and holistic risk formulation that includes the clinician’s attributions within a broader and more meaningful person-centred context. For example, the clinician might deduce that for Client X, injury to high-risk locations reflects their habituation to pain and fear and therefore greater acquired capability for suicide [18]. Yet, risk assessment and formulation are complex, and while it is important to consider whether visual inspections of the wounds fall within the professional’s scope of practice, many non-medically trained health professionals (e.g., psychotherapists, psychologists) encounter self-injury wounds that could affect their judgment [36]. Regardless, therefore, of training and role, understanding whether injury location influences attributions about self-injury is important, as it may affect practitioner-service user interactions [35] and clinical decision-making through inappropriate care.

### 1.3. Aims of the Study

In this study, we addressed an important knowledge gap by using inductive thematic analysis to explore how health professionals construct clinical and psychological meaning based on the location of self-injury on the body, particularly in relation to concealability. We explored three questions:

(1) What do staff think about the clinical risk associated with self-injuring in concealed and visible locations? We speculated that professionals may view cutting near major blood vessels (e.g., the neck) as more clinically risky and associated with suicidal intent, mirroring findings from studies of those who self-injure. We also explored whether staff perceived cutting in visible areas as less clinically risky, potentially because visibility communicates the need for medical attention and support.
(2) What do staff think about the distress experienced when individuals self-injure in concealed and visible locations? We explored whether staff made any location or visibility-based attributions about distress.
(3) What do staff think about the functions of self-injury in concealed and visible locations? We reasoned that staff may be more likely to attribute concealed cutting to intrapersonal (affect regulatory) functions, because the hidden nature of the injury may prevent attributions such as “attention-seeking”. In contrast, staff might assume that injuries in visible areas serve interpersonal functions, since they are physical manifestations of the need for support.

Given the topic’s novelty, we expected a degree of exploration and uncertainty, which we additionally explored through an inductive summative content analysis of the content and style of interview narratives. This analysis provided linguistic evidence by quantifying uncertainty markers (e.g., “might” or “may”) and identifying the frequency of terms used to explore injury location, such as “suicide”. By integrating both thematic analysis and content analysis, we aligned what participants said with the frequency with which they said it.

## 2. Materials and Methods

### 2.1. Recruitment and Sample

We used opportunity sampling to recruit nineteen health professionals between January 2019 and December 2020. There was one inclusion criterion: professional experience in providing support to individuals who self-injure. We did not request a minimum level of experience. The research team shared posters on social media sites such as “X” or via notice boards at a university or in local private mental health services, in North-West England. We selected this sample because the majority who self-injure have not attended the Emergency Room for clinical intervention [7], but may have received support from health professionals in a range of roles, such as mental health support workers, psychologists, psychological practitioners, and counsellors. While each professional has a different role and reason for seeing an individual who self-injures, they all must identify, prevent, and reduce risk.

Table 1 shows the sample characteristics, which included mental health nurses/nurses, support and recovery workers, psychologists, and trainee psychologists. Most participants (80%) identified as female and reported ages between 20 and 59. Just over half received training focusing on self-harm, despite their vocational experiences and broader professional training in mental health. Over 70% had between 1 and 10 years of clinical experience in the sector, and 30% had more than ten years.

Given the novel topic, we aimed to recruit around 20 participants, which we expected might lead to thematic saturation. We also applied the five dimensions of ‘information power’ to determine a sample of 20 [37]: (1) a specific aim, (2) participants of dense specificity, (3) limited theoretical underpinning, given the exploratory and novel topic, (4) less than rich interview dialogue (the interviewer was a Psychology Masters student with some experience of self-injury), and (5) the need for an exploratory cross-case analysis [37].

### 2.2. Ethics

We obtained ethical approval from the School of Psychology Ethics Committee at the University of the first author. Participants provided written informed consent before taking part. We held data securely and confidentially per General Data Protection Regulation (GDPR).

### 2.3. Interviews

The second author conducted semi-structured interviews, either face-to-face or over the phone, with an encrypted recording device. Interviews lasted approximately 30–45 min, with a topic guide and open-ended questions to promote a natural flow of conversation focused on: the locations of self-injury on the body that the individual has come across, the potential meaning (if any) location might have for those who self-injure with regards to risk, emotional distress, and self-injury functions, particularly in relation to visible and concealed self-injury.

### 2.4. Analytical Strategy

This study adopted a critical realist perspective, using qualitative, reflexive inductive thematic analysis of interview data and following Braun and Clarke’s [38] steps. This process allowed us to identify recurring themes within and across participants. We had achieved thematic saturation when new codes reflected only small variations rather than substantive new themes.

The analysis consisted of six successive phases: Several of the core research team ensured thorough familiarization with the raw data (Phase 1), to become immersed and engaged via a joint data management process. The team then meticulously reviewed all raw data to generate initial codes (Phase 2), grouping codes for organizing themes into each topic area. The core research team coded 25% of transcripts, meeting repeatedly to review, discuss, debate, resolve agreements and reorganize codes into key themes (Phase 3). We then jointly described and discussed each theme to ensure consensus before sharing the final set of themes with two extended members of the team for review (Phase 4). Several reviews and iterations of themes aided the core and extended research team, to re-define and then re-name the themes (Phase 5), resulting in a final set of themes (Phase 6).

To ensure the transparency and credibility of data, the interviewer kept a reflexive journal, allowing a personal record of comments and potential biases. The team also reflected on how their knowledge and their own beliefs about self-injury may have shaped the analysis. The team had different backgrounds in psychology/nursing, varied knowledge of or experience working with self-injury (academic or clinical), and varied skills in thematic analysis (novice or experienced), leading us to explore varied perspectives and interpretations, while staying true to participants’ voices.

We used inductive content analysis to supplement the thematic analysis, as a flexible approach to analyzing text data generated by the interviews [39]. This analysis shifted the focus to quantifying language patterns and word usage/frequency. We followed Tausczik and Pennebaker’s [40] categorization by exploring content (i.e., what staff were most commonly focusing on when exploring location) and style (i.e., how staff were talking about location, such as whether they expressed uncertainty when addressing the issues). NVivo [41] word frequency query listed the most frequent words in the transcripts, after reducing noise in the data by removing insignificant ‘stop words’ (e.g., ‘and’), and limiting results to words of three letters or more. We also applied text match settings to our query criteria, which allowed us to find exact matches and group words with the precise stem together. A first pass through the data prompted us to search beyond exact matches and identify similar words and synonyms, which we grouped to find the most frequently occurring concepts. For example, we grouped ‘risk’ with ‘chance, danger, dangerous, dangers, risk, risks’.

## 3. Results

### 3.1. Inductive Thematic Analysis

This paper reports on seven themes, as shown in Table 2. Example direct quotations illustrate each theme.

#### 3.1.1. Theme 1: Location Drives an Appraisal of Risk

This subtheme included staff experience of assessing and evaluating the risks associated with self-injury, such as infection, fatality, and suicidal intent. There was recognition amongst the majority that some locations may be “more high risk to harm than others” (P4) or fatal, including areas with major blood vessels, the neck, and face. Staff discussed suicide risk, with some suggestion that self-injury to these locations shows an intent to end one’s life. Others suggested that “they’re not aware of the risk” (P12) associated with self-injury in high-risk locations, and that service users’ mental health and levels of distress can affect their self-assessments of risk. Alternatively, avoidance of ‘high-risk’ locations could reflect conscious risk awareness.

Some staff described that concealed locations increase the risk of infection (P9,16) or death/suicide (P16,19), since it hinders monitoring. Visible locations might decrease risk (P2,5,20) because they enable identification and management.

#### 3.1.2. Theme 2: Driven by Emotion and Selecting a Location of Relief: A Location-Based Perspective of the Intrapersonal Functions of Self-Injury

This subtheme captured how staff may attribute injuries in certain locations to emotional relief and affect-related factors. Some staff believed that areas more sensitive to pain provide greater relief, but that service users may move locations as a “tolerance to pain develops” (P8).

There was a sense that intrapersonal difficulties might underpin specific locations. For example, some staff discussed how injuries to the face or neck might reflect heightened distress, and Participants 9, 10 and 14 described experiences of supporting those with facial injuries that signified underlying feelings of self-loathing, guilt, shame, or self-punishment. Cutting self-identifying words such *as* “fat” into the stomach or thighs was believed to reflect dislike of that area, whilst acting as a visual reminder for the person (P18).

Many staff described how service users may injure concealed locations to “release” (P1, 11, 12, 16, 17) or “regulate” (P15, 16, 18) emotions and distress (i.e., intrapersonal functions). Staff felt that concealed locations reflect privacy, secrecy, shame, and self-punishment (P1, P2, P4, P6, P12, P13, P18). Participant 10 also described how injuries to concealed areas might reflect more cognitive control: “more awareness and more of a thought process behind it in terms of taking that time to conceal it and choosing a specific location….when it’s not concealed, I think it’s more impulsive…”.

Interestingly, there were conflicting beliefs about how distress might relate to injury location. Some thought concealed locations might reflect more heightened distress than visible areas, and others suggested the opposite. Some staff took the view that distress is the same irrespective of location and emphasized the importance of a person-centred approach: “the majority of the time…it kind of just depends on the context of the incident regarding distress…it really does vary person to person” (P2).

#### 3.1.3. Theme 3: Selecting a Visible Location for Interpersonal Reasons

Just over half of the staff believed that visible injuries could help meet interpersonal needs, such as communicating emotions or eliciting a care-seeking response or “attention” (P4, 5, 8, 13, 14, 15, 17). For example, when discussing visible locations including the face, arms and neck, some staff suggested that service users “want us to know they’re frustrated” (P1) and “want the attention” (P5), though, some staff acknowledged that distress also underpins visible self-injury.

#### 3.1.4. Theme 4: Contemplating the Role of Demographic Factors, Mental Health Diagnoses and Wider Experiences

Some staff explored mental health diagnoses, wider experiences such as trauma and social media, and demographic factors such as gender. Many described how service users with past traumatic experiences might injure “where that abuse has taken place” (P10), such as to the tops of the thighs, breast, or genitals, in the case of sexual abuse. Yet, staff offered explanations other than physical or sexual abuse for injuries to the breast and genitals, such as being unsure about one’s gender. Some staff also believed that mental health diagnoses might explain certain injury locations. For example, they linked abdominal injuries to eating disorders due to body fat, and facial injuries to psychosis and a loss of control.

A few staff noted that observing others’ injuries could influence location choice, as some sites elicit “a massive response... [due to] how dangerous the location is” (P1). Others suggested that exposure via social media played a role in location choice, especially for younger adolescent females who model what they see (P8).

#### 3.1.5. Theme 5: A Pragmatic Perspective of Location

Staff described potential pragmatic drivers of location, including accessibility, ease of access, convenience, right/left-handedness, physical sensation, and unscarred skin. Some suggested that scar tissue from earlier self-injuries might influence location: “they’re looking for that new feeling” (P8) and the “damage is already done to different parts of the body” (P10). Some staff explored how locations such as the arms and hands are accessible (P5, 9, 17, 19) and convenient (P1, 13, 19), as “it’s easier to pull your sleeve back and cut your arm... [than] stomach” (P9). Additionally, self-injury in accessible and convenient locations may reflect an impulsive decision to injure “wherever they can access” (P17)*,* such as banging their head against a wall when in hospital without tools (P1, 16). Finally, Participant 12 believed that biological handedness, such as injuring “the left arm because they’re right-handed” might play a role.

#### 3.1.6. Theme 6: Location and the Bigger Picture

Some staff considered other contextual factors and clinically presenting features alongside location when making sense of self-injury and evaluating risk. In essence, “It’s not just the location, it’s the nature of the self-harm as well” (P4). Some believed that it’s important to focus more on severity (P3, 17) of the self-injury, including the depth (P4, 8, 17) and direction of the cuts (P8, 17), regardless of location. Some staff discussed how they attend to the type of harm and method when addressing self-injury, implying that some methods may be riskier than others, irrespective of location. However, many of the same staff either suggested or implied that location was an important factor in self-injury, highlighting a degree of uncertainty as to the significance of location.

#### 3.1.7. Theme 7: The Impact of Injury Location on the Staff Supporting Individuals Who Self-Injure

The emotional and physical impact of injury location on staff emerged throughout the narratives. For example, some discussed how they have become “desensitised” (P18, 19) to locations including the wrists and arms, with less common areas such as the genitals, face and neck being more distressing, shocking, and unmanageable for staff. Some felt that concealed injuries were difficult for them to manage, appearing to be a catalyst for feelings of helplessness for staff and the perpetuation of stigma. Staff described visible injuries or scars as attracting stigmatizing views, including from members of the public. Participant 15 suggested, however, that self-injury alone may trigger a panicked response for new and less experienced staff, regardless of location.

### 3.2. Inductive Summative Content Analysis

**Figure 1:**
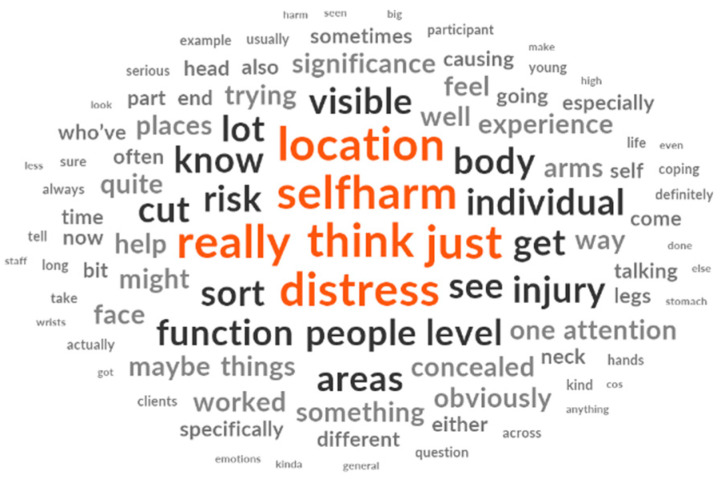
Word cloud (graphical representation of word frequency) produced from the interviews with participants’ corpus, displaying up to 100 words.

As expected, “self-harm” was the most frequently occurring content word and interview topics were common (e.g., “location”, “distress”, “function”, and “risk”), as shown in Table 3 and Figure 1. However, “cut” was also common, even though we did not enquire about self-injury method during the interviews. Style words were common, including insight words (a type of cognitive mechanism) such as “think” and “know” and more complex terms greater than six letters, such as “individual”. Staff frequently used the word “individual”, likely reflecting a reliance on individual cases as they explored broader issues and navigated certain assumptions. Finally, tentative language such as “might” and “just” was common, highlighting an uncertainty in the narratives.

## 4. Discussion

To our knowledge, this is the first study to show that staff attribute meaning to the location of the self-injury on the body. We identified seven themes. Staff explored the clinical significance of injury location in Theme 1, which focused on location-based appraisals of risk, and Theme 6, which positioned location as one of multiple factors considered when making sense of self-injury and evaluating risk. Themes 2–4 captured the perceived psychological meaning of location, addressing intrapersonal functions and distress relief (Theme 2), interpersonal functions, support-seeking, and visibility (Theme 3); and wider psychosocial factors (Theme 4). Pragmatic drivers of location were also considered (Theme 5), along with the impact on staff (Theme 7). Conflicting views and tentative language highlighted uncertainty and interpretative ambiguity around the significance of injury location. Some discussed the importance of an individualized approach to self-injury, but location-based generalized attributions were common and may not align with the lived experience of self-injury, as we discuss below.

Regarding clinical significance, staff views about risk (Theme 1) generally align with anatomical realities and previous research, as staff viewed injuries to the neck as higher risk and life-threatening [19]. These views perhaps reflect an awareness that injuries to certain areas reflect the individual’s acquired capability for suicide [18], which they may or may not be consciously aware of. In addition, the view that concealed locations are higher risk aligns with a study that has found associations between concealed self-cutting and factors such as premeditation and repeat self-injury [21]. Ultimately, staff had heightened concerns about the potential for life-threatening, unnoticed self-injury. Considering injury location when assessing the severity of self-injury could direct “a more intrusive clinical and medical response” [42] (p. 383), though studies have yet to explore this.

Staff perspectives on the psychological significance of location reflect an interpretive and diagnostic lens, linking specific injury locations to underlying psychosocial factors, experiences, and disorders (Themes 2–4). Diagnostic framing of injury location (e.g., psychosis may drive facial self-injury and eating disorders may determine stomach injuries), is a generalized attribution that pathologizes specific injury locations, and these attributions may not align with the individual’s lived experience. Moreover, visibility-based assumptions—such as concealed self-injury reflect intrapersonal emotion regulation and visible locations reflect interpersonal communication—mirror beliefs expressed by adolescents [32,33]. Such interpretations resonate with existing theory on how the functions of self-injury may relate to injury location [15], and they may also reflect an individual’s genuine motivations for self-injury [20]. However, it is important to adopt a person-centred approach by asking the individual if injury location has such meaning for them, since generalized attributions triggered by visible injuries may hinder validation of an individual’s internal distress and weaken the therapeutic alliance, leading to subtle, unconscious changes in staff behavior. This may be especially true if staff refer to visible injuries as an attempt to seek “attention”. This is a pejorative and dismissive term that can imply something is wrong with the individual, reinforcing shame and stigma and damaging trust, leading the service user to disengage from the staff member providing care. The term is not in keeping with Trauma-Informed Care (TIC), which views self-injury as a behavioural coping strategy and expression of unmet needs in response to intense emotions and past or ongoing trauma [43,44]. Similarly, generalized attributions triggered by concealed injuries can conflate concealment with secrecy and shame, potentially obscuring an individual’s internal struggles and influencing clinical engagement or leading to inaccurate risk assessment and formulation. Not all staff held the same visibility-based views, however, with some emphasizing a person-centred approach and others appearing uncertain and speculative as they constructed meaning around injury visibility.

The pragmatic perspective (Theme 5) provided by some of the same staff resulted in a notable juxtaposition with clinical and psychological factors (Themes 1 to 4), offering a simplified view that strips location of clinical, affective, and interpersonal meaning. While staff did not explicitly link pragmatic drivers to suicide risk, repeatedly injuring locations due to convenience and accessibility could nonetheless contribute to increased pain tolerance in this region and thus an acquired capability for suicide, a key element in Joiner’s theory [18]. Some staff highlighted the change in location as tolerance to pain develops (Theme 3), although they did not conceptualize this as contributing to an acquired capability for suicide.

Location was often a starting point for understanding self-injury that must be considered alongside important clinical and contextual factors that are known to elevate risk (Theme 6), such as method, severity of the injury and the damage to the individual (Theme 6). These reflections highlight a person-centered practice approach to understanding a complex behaviour based on a multitude of presenting clinical features. Participants acknowledged that solely focusing on location may lead staff to miss other significant factors that might elevate the risk, such as method and severity [45,46]. It is noteworthy that the content analysis found “cut” to be frequently mentioned over other methods, such as burning, suggesting that staff held cutting in mind when answering questions, perhaps reflecting that self-cutting is a common and stereotypical method of self-injury.

Finally, strong reactions to self-injury are common and widely acknowledged [47], yet reactions such as shock, panic and trauma in response to more visible and/or “shocking” areas, such as the face (Theme 7) highlight a heightened emotional response and the vulnerability of this workforce. The emotional impact on staff working with self-injury is well-documented, with studies showing adverse effects on their capacity to provide care, including reduced engagement and withheld referrals [48,49]. Similarly, staff’s adverse emotional experiences open the potential for them to experience stigmatizing views that can reduce empathy and lead to lower-quality care as staff distance themselves as a form of self-preservation and struggle to balance personal emotional reactions, with compassion and a non-judgmental attitude. These emotions are automatic and natural responses to distressing events, but they should be managed to prevent biased assessments and formulations, as well as adverse impacts on care, such as delayed referrals, misaligned interventions, or excessive control measures that restrict service user agency as a form of risk management.

### 4.1. Limitations and Future Directions

This study has limitations. First, while the higher proportion of female professionals (80%) recruited from social media and local services reflects the prevalence of females in some of these professional roles (e.g., psychological services [50]), it may reflect self-selection bias. Females hold comparatively more favorable attitudes towards self-injury than males [51], which may motivate study participation. Similarly, recruiting through social media networks may limit the generalizability of our findings to the broader population because individuals with strong opinions on the subject may have self-selected into the study. Future studies should use a stratified sampling approach across demographic and professional subgroups to reduce self-selection bias and broaden the diversity of perspectives.

Second, varied professional roles and experiences, such as indirect or direct exposure to self-injury, provide diversified perspectives and attributions. Some staff also drew on others’ experiences, such as their colleagues, as they ascribed meaning. These vicarious experiences provide a broader perspective but may lead to some narratives receiving undue emphasis. Diverse roles and experiences also enrich the data and reflect the nature of multidisciplinary teams that support those who self-injure. Hence, the findings are transferable to other clinical settings [52] where staff work with those who self-injure. Understanding both developing and experienced perspectives is also key to providing effective client care. Future studies should report the proportion of participants who had personally witnessed self-injury and how recently (e.g., within the last 30 days), to contextualize reflections. Studies should also recruit sub-groups of health professionals with, for example, different levels of training in risk assessment and direct or experience of conducting visual inspections of self-injury wounds within their scope of practice [36].

Third, enquiring specifically about concealed and visible self-injury may have encouraged staff to think in dichotomous terms, though some staff challenged the visible-concealed categorization with a more complex and nuanced understanding of self-injury. Moreover, in adopting a critical realist perspective, we acknowledged that the interviewer’s limited experience in the field may have reduced the depth of the participants’ responses (e.g., by missing nuances in the participants’ responses and not following these up). Yet, the wider team’s extensive knowledge, expertise, experiences, and theoretical positioning further shaped how we interpreted the narratives. Staff reflections on self-injury location, as well as our interpretations of these data, may have differed from a more culturally and ethnically diverse sample and research team. However, Gardner et al. [15] note that injury location tends to be relatively consistent across both Western and non-Western cultures.

Future research should address the above limitations and quantitatively examine whether injury location influences clinical interactions and decision-making, including risk assessment and formulation. It is important to explore lived experience perspectives to understand the personal reasons individuals choose to injure specific body locations, and how these reasons align or diverge from staff perceptions and attributions (such work is currently underway by the first author). It is possible that some of our sample also had personal experiences of self-injury that shaped their perspectives.

### 4.2. Clinical and Educational Implications

**Figure 2:**
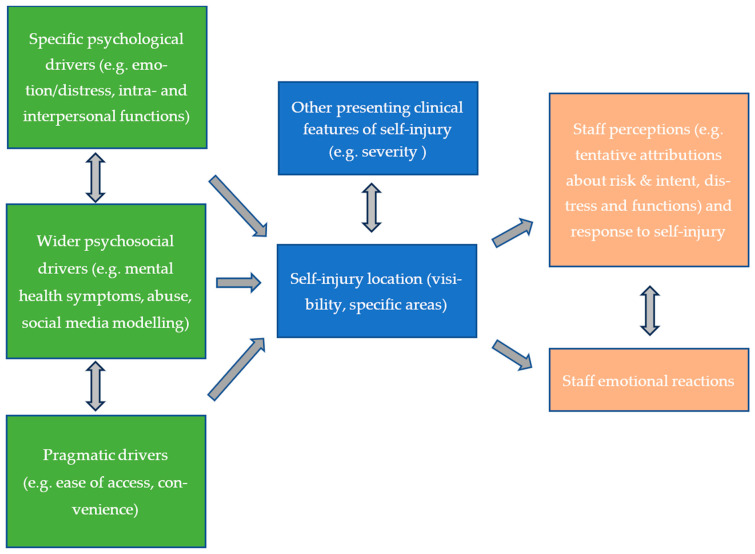
Conceptual model illustrating health professionals’ views on perceived lived experience factors (green panel; Themes 2–5) that may jointly influence the location of self-injury (lower blue panel). When considered alongside other clinically presenting features (upper blue panel), these factors can affect how staff respond (upper orange panel; Themes 1, 6) and react (lower orange panel; Theme 7), with both influencing each other.

A key clinical recommendation is adapting self-injury training based on our findings (Figure 2). Evidence-based self-injury and suicide prevention training, such as ‘Skills Training on Risk Management (STORM), delivers psychoeducation and equips staff to use a person-centred approach, building their skills in engagement, psychosocial assessment, collaborative formulation, and safety planning [53]. Training programs such as STORM significantly improve staff knowledge, confidence, attitudes, and skills [31,54,55]. We recommend that such training be adapted to consider injury location, to increase understanding of location, raise awareness of related attributions, and enhance the development of key skills. Given the elevated risk of suicide [3,4,5] and economic costs associated with self-injury [56,57], staff training and organizational support in this area is an important endeavor.

#### Recommended Adaptations to Self-Injury and Suicide Prevention Training

(1) Communication, engagement, and relational skills: Training should encourage a non-judgmental, compassionate, person-centred, and respectfully curious approach [58,59,60], irrespective of injury location. Staff should begin by building a rapport, pacing the conversation, and giving space to talk before gently beginning to explore location and other aspects of the self-injury using open-ended, supportive questions such as: “How would you like me to support you when we talk about injuries to [location X], and [location Y]?” It is conceivable that injuries in specific areas may be more difficult to talk about than others, particularly those that elicit shame or perceived stigma; hence, staff should be attuned to signs of discomfort and ensure the service user feels reassured and supported.
(2) Collaborative and individualized clinical assessment skills: Staff should be trained to work jointly with service users to assess each injury episode [5] and understand whether injury location holds psychological or clinical significance for the individual. The assessment may include visually inspecting wounds, if this falls within one’s professional scope of practice [36]. Staff should adopt a stance of respectful curiosity [59,60] and might ask the service user: “What, if anything, does this injury location mean to you?”
(3) Collaborative risk formulation and integration skills: Staff should be trained to incorporate injury location into individualised, dynamic, and holistic collaborative risk formulations [6,61] that empower service users and help them understand the risk posed by their self-injury in specific locations, explored in relation to their history, current difficulties, and context. Staff should embed the questions within this wider conversation and might begin: “I’m also curious about how you feel just before you injure location X?... and whether injuring this area feels more dangerous or more significant to you?”
(4) Reflective practice and critical thinking skills: It is essential that training develops awareness of the potential for implicit biases and location-based perceptions, assumptions, and attributions (e.g., “visible injuries always indicate a communicative function or lower risk”) that might need challenging to avoid stigma or unintended clinical consequences, such as inaccuracies in risk assessment and formulation. This is in keeping with a trauma-informed approach to care, which can significantly reduce self-injury [44]. Staff could be trained to use the six stages of Gibbs’ reflective cycle [62] to ask themselves questions: (1) Description, “what did I see (e.g., facial self-injury)?”; (2) feelings, “how did I feel when I saw this injury”; (3) evaluation, “how helpful/unhelpful was my response?”; (4) analysis, “did I make any assumptions about this injury, and were these shaped by where the injury is on the body?”, and “did these assumptions affect my response or decision-making?”; (5) conclusion, “what else could I have done?” and (6) action plan, “what strategies can I use to manage by emotional responses if I see injuries on location X again?...what support do I need to help me provide the best care?”
(5) Self-care and emotion management skills. Staff should be supported to understand and manage automatic, intense emotional reactions, such as shock, that can arise when encountering injuries, particularly in sensitive or less typical locations. These emotional reactions impact staff well-being and should also be considered through a relational lens, encouraging reflection on how different responses might influence interactions with service users. Training should cover professional self-care strategies [23] and the development of emotion regulation techniques, such as grounding and mindfulness. Equally important is the provision of role-specific organizational support structures to help staff process their emotions constructively. This might include regular supervision, team debriefs following distressing incidents, peer/colleague support, and reflective practice.

## 5. Conclusions

This paper is the first to explore whether the location of self-injury on the body holds clinical or psychological meaning for health professionals who provide care. The findings show how staff understand this deeply complex and individualised behaviour. While staff attributions about risk may align with studies showing that suicide risk can increase for locations such as the neck, staff should avoid generalized location-based attributions about risk or aspects such as distress and self-injury function. Staff should approach each episode of self-injury as unique, using a stance of respectful curiosity whilst collaboratively exploring the personal significance of location for each episode of self-injury [6,59,60]. Training for those working with individuals who self-injure should consider injury location to increase understanding, raise awareness of related attributions, and improve the development of clinical skills. This includes conducting individualized, dynamic, and holistic risk formulations that integrate the meaning of location. Organisations should support staff in this endeavor, implementing support structures such as reflective practice to help staff manage the emotional impact of working with individuals who self-injure and who are at risk of suicide. Future research should investigate whether location-based attributions are associated with unintended clinical consequences, such as inaccuracies in risk assessment and formulation.

## Figures and Tables

**Table 1:** Demographic features of participants.

	Frequency	Percentage
Gender		
Female	15	79
Male	4	21
Age		
20–29	8	42
30–39	5	26
40–49	4	21
50–59	2	11
Qualification		
BTEC Diploma	1	5
Bachelor’s Degree	2	11
Master’s Degree	12	63
PHD	1	5
Missing	3	16
Job Role		
Support Worker	4	21
Recovery Worker	1	5
Mental Health Support Worker	3	16
Nurse	1	5
Mental Health Nurse	1	5
Trainee Counselling Psychologist	1	5
Trainee Forensic Psychologist	5	27
Psychologist	1	5
Consultant Forensic Psychologist	2	11
Years of Clinical Experience		
<1	1	5
1–2	2	10
3–4	4	21
5–6	2	11
7–8	3	16
9–10	2	11
>10	5	26
Self-Harm Training Undertaken		
Yes	11	58
No	8	42

**Table 2:** Themes.

Themes
(1) Location drives an appraisal of risk (2) Driven by emotion and selecting a location of relief: a location-based perspective of the intrapersonal functions of self-injury (3) Selecting a visible location for interpersonal reasons (4) Contemplating the role of demographic factors, mental health diagnoses and wider experiences (5) A pragmatic perspective of location (6) Location and the bigger picture (7) The impact of injury location on the staff supporting individuals who self-injure

**Table 3:** Top 25 most frequently occurring words throughout the interviews.

Word	Length	Count	Weighted Percentage (%)
think	5	808	3.88
self-harm	8	552	3.17
distress	8	443	1.98
location	8	421	1.97
just	4	363	1.83
really	6	339	1.63
people	6	274	1.58
see	3	467	1.46
risk	4	267	1.44
individual	10	309	1.40
sort	4	314	1.39
function	8	398	1.34
level	5	294	1.34
body	4	234	1.32
know	4	310	1.13
get	3	493	1.12
areas	5	192	1.10
visible	7	190	1.09
injury	6	253	1.08
cut	3	197	1.05
lot	3	240	1.02
concealed	9	218	1.01
well	4	180	0.97
might	5	167	0.96

## Data Availability

The data supporting this study are not publicly available due to the sensitive nature of the topic. The research team restricts access to the data and will not grant it to ensure confidentiality and ethical compliance.
